# The Effect of Maternal Intact Protein- and Amino Acid-Based Diets on Development of Food Intake Regulatory Systems and Body Weight in Dams and Male Offspring of Wistar Rats

**DOI:** 10.3390/ijms20071690

**Published:** 2019-04-04

**Authors:** Alireza Jahan-mihan

**Affiliations:** Department of Nutrition and Dietetics, University of North Florida, 1 UNF Dr., Jacksonville, FL 32224, USA; alireza.jahan-mihan@unf.edu; Tel.: +1-904-620-5359

**Keywords:** protein, amino acid, fetal programming, food intake, appetite, body weight

## Abstract

The objective of this study is to examine the effect of maternal and weaning intact protein- and amino acid-based diets on regulation of food intake, intake regulatory hormones, and body weight in dams and their male offspring. Pregnant Wistar rats were allocated to two groups (*n* = 12) and were fed either an intact protein diet (IPD) or mixed amino acid diet (AAD) from day 3 of gestation throughout gestation and lactation. Male offspring were weaned to either an IPD or AAD for 18 weeks. Food intake (FI) and body weight (BW) were measured weekly. Results: In dams, the AAD group had lower FI and BW in the post-partum period compared with the IPD group. In pups born to AAD dams, birth weight and BW were lower. However, the percentage of fat and lean mass were not affected. Food intake was influenced by maternal diet and was higher in pups born to IPD dams throughout post-weaning. Short-term FI in response to protein preloads was lower in pups born to AAD dams in 1 h. Fasting plasma concentrations of glucose, insulin, and ghrelin were not influenced by either maternal or weaning diet. However, peptide YY (PYY) was higher in pups born to IPD dams at weaning. Conclusions: The physicochemical properties of proteins fed during pregnancy and lactation had determining effects on the body weight and development of food intake regulatory systems in offspring. Maternal AAD resulted in lower BW in dams and lower birth weight and post-weaning BWs in pups compared with maternal IPD which was consistent with their lower FI.

## 1. Introduction

Dietary proteins fed during gestation, lactation or both alters the phenotype of offspring in humans and animals [1]. It is suggested that the source of proteins in maternal diets can also be a factor influencing the development of metabolic and physiologic regulatory systems in offspring [1].

Both high- and low-protein maternal diets showed detrimental effects on body weight, body composition, and food intake regulatory systems in the offspring [2,3,4,5,6,7,8,9,10,11,12]. Source of protein in maternal diets can also be a factor [1]. We previously reported that pups born to dams fed a soy protein-based diet had higher body weight (BW) at 14 weeks post-weaning compared with those born to dams fed a casein-based diet [13]. Food intake (FI) was also higher in offspring born to dams fed a soy protein diet in the post-weaning period [14,15]. Proteins in a weaning diet may also interact with maternal diet. In rats, offspring born to dams fed a low-protein diet and weaned to corn and soy protein-based diets had lower weight and body fat percentage compared with those born to dams fed a low-protein diet and weaned to a casein-based diet. Therefore, source of protein in the weaning diet can also be a factor influencing the outcomes of maternal protein-restricted diets [16].

The effect of proteins on satiety, food intake, body weight, and composition is source-dependent [3]. Metabolic and physiologic properties of each protein are unique and are determined by factors that are structure-dependent. Amino acid composition, digestibility, digestion kinetics, bioactive peptides encrypted in amino acid sequence, and finally non-protein bioactive components conjugated with proteins are dependent on the structure and physicochemical properties of proteins and are major factors determining metabolic and physiological response to each protein [3]. The difference in amino acid composition of proteins determines their effect on body functions [17].

Based on amino-static theory, incomplete proteins have greater suppression effect on appetite than complete proteins [18]. It is based on this belief that the primary function of proteins is protein synthesis and the remaining amino acids (AAs) in circulation can act as satiety signals [18]. The effect of individual AAs on satiety, food intake, and protein synthesis has also been studied. The satiety effect of tryptophan and also the tryptophan/large neutral amino acid ratio through their effect on serotonin, a neurotransmitter that enhance satiety sensation, is reported [19]. Moreover, intracerebroventricular administration [20] and also increased dietary intake of leucine [21] decreased food intake. The satiety effect of tyrosine through synthesis of dopamine [22] and histamine has also been reported [23]. The effect of individual AAs on body composition is also shown. For example, the stimulating effect of branched-chain AAs, particularly leucine, on protein synthesis is evident [24].

Digestibility of proteins is also a factor that determines postprandial amino acid availability [17]. Animal proteins generally have higher digestibility compared with plant proteins [25]. Moreover, the difference in digestion kinetics of proteins may result in variation in their postprandial metabolic effects that can affect protein synthesis and satiety [17]. For example, casein is classified as a slow protein and results in a slower and more persistent amino acid flow to the gut and eventually to the blood compared with whey protein that rapidly passes through the stomach and causes a faster delivery of AAs to the circulation in a shorter period of time. It leads to a larger increase in post-meal aminoacidemia than casein resulting in a higher protein synthesis, whereas casein has a greater effect on lowering protein breakdown [3].

The source-dependent effect of proteins on developmental programming observed in previous studies supports the potential role of the characteristics of individual proteins in their metabolic and physiologic outcomes. We previously reported that casein and soy protein, as the sole sources of protein in the maternal diet, influenced the phenotype of the offspring differently in Wistar rats [13,14,15]. In another study, offspring born to dams fed with a low-protein diet and weaned to corn and soy protein diets had lower weight and body fat percentages compared with those born to dams fed a low-protein diet and weaned to a casein-based diet, indicating that the source of protein in the weaning diet can alter the outcomes of maternal protein-restricted diets [16]. There is a line of evidence supporting the source-dependent effect of proteins consumed during pregnancy and lactation on the health of offspring. First, in addition to their role in protein synthesis, AAs have their individual effects on fetal and early post-natal development. For example, low-protein diets with similar protein content but different in methionine content (the Southampton diet has higher methionine content compared with the Hope farm diet) affect the programming of blood pressure in the offspring differently [1]. The Southampton maternal diet with higher methionine content resulted in higher blood pressure in offspring compared with the Hope farm maternal diet. Adding glycine to the Southampton diet, which reduces plasma homocysteine, leads to normalization of blood pressure. This may suggest that the methionine load is a contributing factor to increased blood pressure in offspring [26]. Moreover, taurine supplementation can regulate insulin secretion and promote insulin sensitivity. Taurine can also ameliorate fructose-induced hyperglycemia, hypertension, and hepatic steatosis in pregnant and non-pregnant rats [27].

In addition to the effect of individual AAs, a great number of physiological functions of proteins are attributed to their bioactive peptides (BAPs) [28]. For example, BAPs inhibit angiotensin converting enzyme (ACE), which lowers blood pressure in experimental animals [29]. Casomorphins are one of the major groups of BAPs that are abundant in casein and affect food intake regulation, gastro-intestinal movement, and plasma insulin concentrations [30,31,32]. However, whether BAPs contribute to the effect of maternal proteins on developmental programming is still elusive.

This is the first study, to the best of our knowledge, that examined whether the effect of maternal dietary proteins on fetal development is attributed to the individual AAs or those characteristics of proteins that are dependent on their structure and physicochemical properties (e.g., digestion kinetics, BAPs encrypted in amino acid sequence, amino acid composition, non-protein bioactive components conjugated with proteins). The primary objective of this study was to examine the hypothesis that intact protein- and amino acid mixture-based maternal diets fed during gestation and lactation influence the risk of development of characteristics of metabolic syndrome (MS) in the offspring differently. Additionally, to examine the potential main and interactive effects of maternal and weaning diets, the second objective was to determine the effect of the characteristics of proteins of the weaning diet on the outcomes of the maternal diet in the offspring.

## 2. Results

### 2.1. Dams

**Figure 1:**
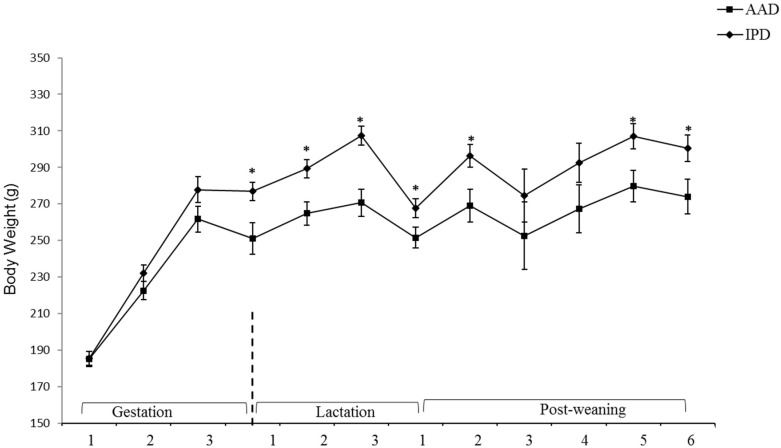
Effect of maternal diet on dams’ body weight (*n* = 12/group). Data are means ± SEM; BW was analyzed by one-way ANOVA. * *p* < 0.05; AAD: amino acid-based diet; IPD: intact protein diet; SEM: Standard error mean; BW: Body weight.

**Figure 2:**
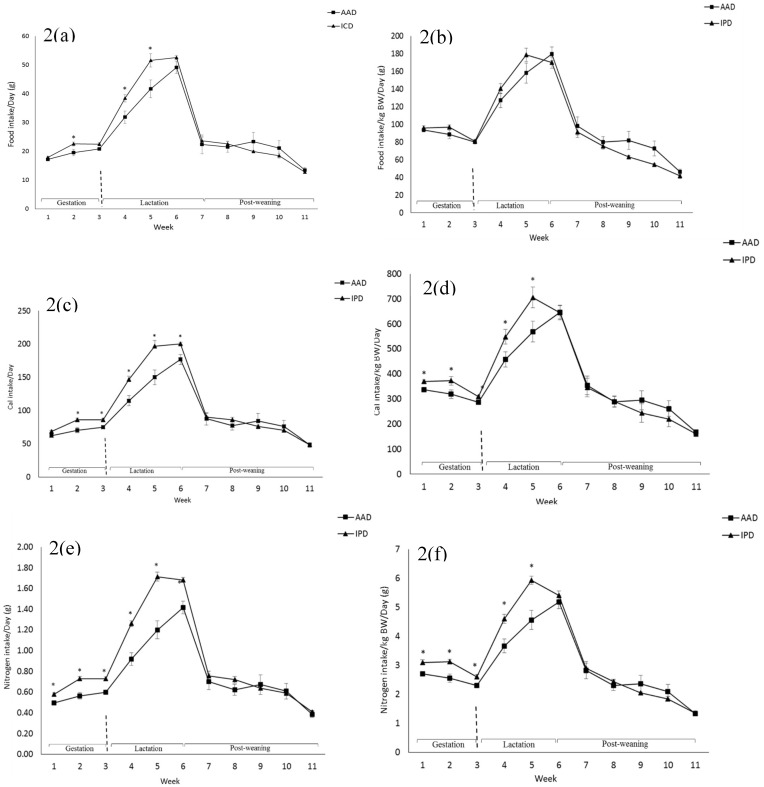
Effect of maternal diet on dams’ food intake (**a**); food intake/kg BW (**b**); Cal intake (**c**); Cal intake/kg BW (**d**); nitrogen intake (**e**); nitrogen intake/kg BW (**f**). (*n* = 12/group). Data are means ± SEM; the intake was analyzed by one-way ANOVA. * *p* < 0.05. AAD: amino acid-based diet; IPD: intact protein diet; Cal: Calorie.

**Figure 3:**
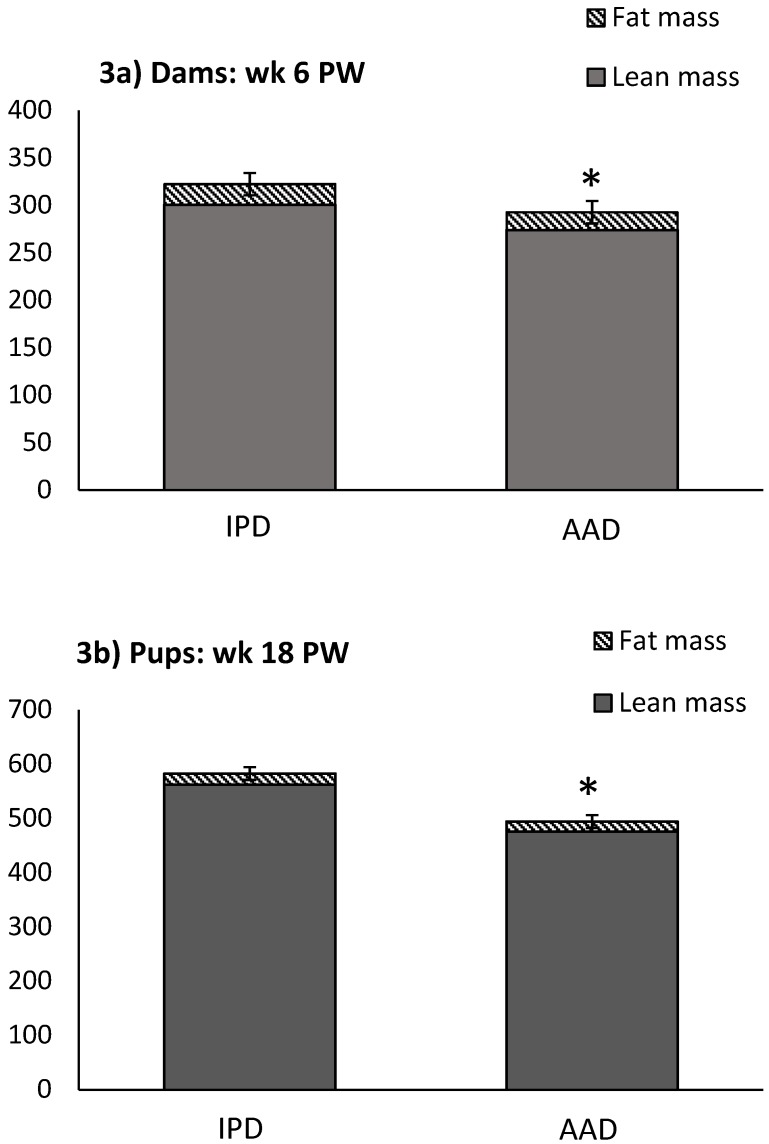
Effect of maternal diet on BW, lean mass, and fat mass in dams at week 6 pw and in pups at week 18 pw. (*n* = 12/group). Data are means ± SEM; * *p* < 0.05. AAD: amino acid-based diet; IPD: intact protein diet. Fat was calculated as the sum of abdominal, epididymal, and prerenal fat. PW: post-weaning.

BW was affected by the diet after parturition, during lactation, and at weeks 2, 5, and 6 pw and was higher in dams fed the IPD (Figure 1). Long-term food intake was also affected by the diet at week 2 gestation, and weeks 2 and 3 lactation. It was higher in IPD dams (*p* < 0.05) (Figure 2a). Consistently, the calorie (Cal) intake was also affected by the maternal diet and was higher in IPD dams from week 2 gestation to the entire lactation period (Figure 2c). Nitrogen intake was also higher in IPD dams from birth to week 2 lactation (Figure 2e). However, while food intake/kg BW was only relatively higher in IPD dams (Figure 2b), both Cal (Figure 1d) and nitrogen intake (Figure 2f), when adjusted based on BW, were still higher in IPD dams throughout gestation and lactation. Fat and lean mass were not affected by maternal diet at week 6 pw in dams (Figure 3a). No effect of the maternal diet on fasting plasma concentrations of glucose (FBG), insulin, ghrelin, and PYY was observed. (Table 1).

### 2.2. Pups

**Figure 4:**
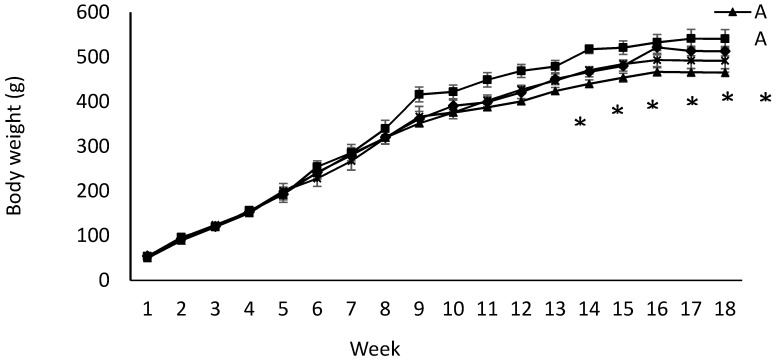
Effect of maternal and weaning diets on post-weaning body weight of male offspring (*n* = 12/group). Data are means ± SEM; BW was analyzed by the Mixed model with maternal diets and time as the main factors. Time: *p* < 0.0001; maternal diet: *p* < 0.009; weaning diet: *p* < 0.06. AA: offspring born to dams fed an amino acid-based diet and weaned to an amino acid-based diet; AI: offspring born to dams fed an amino acid-based diet and weaned to an intact protein diet; IA: offspring born to dams fed an intact protein diet and weaned to an amino acid-based diet; II: offspring born to dams fed an intact protein diet and weaned to an intact protein diet; * Significant effect of maternal diet on body weight: pups born to dams fed IPD (groups II and IA) had higher body weight compared with those born to dams fed AAD (groups AA and AI).

**Figure 5:**
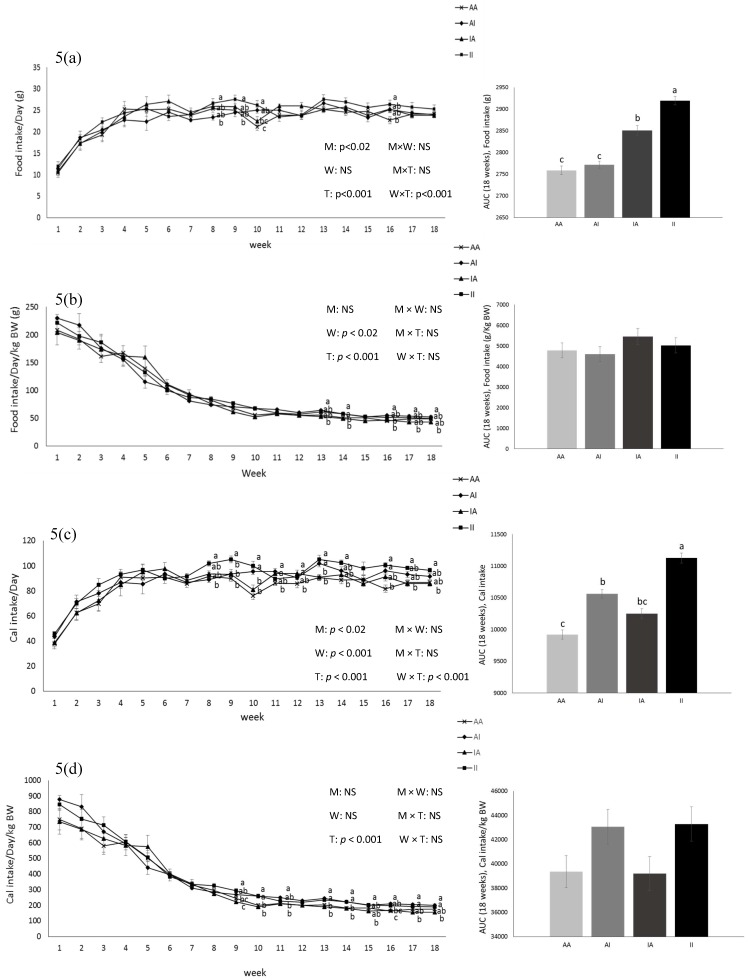

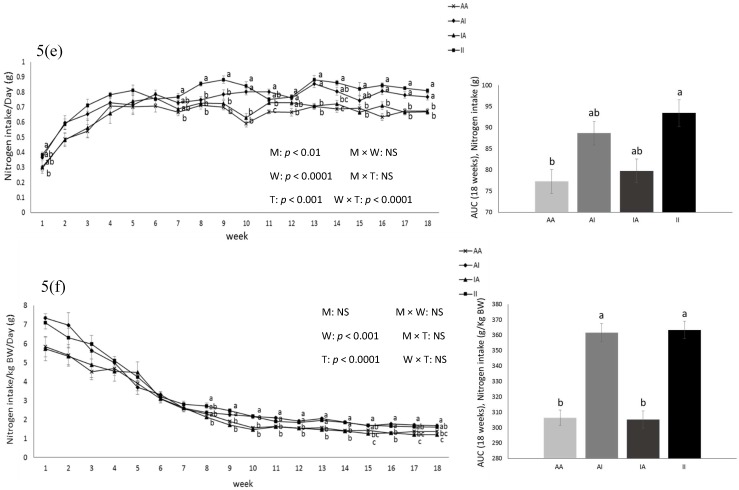
Effect of maternal diet on post-weaning food intake (**a**); food intake/kg BW (**b**); Cal intake (**c**); Cal intake/kg BW (**d**); nitrogen intake (**e**); nitrogen intake/kg BW (**f**) and their area under the curve (AUC) (*n* = 12/group) in male offspring. Data are means ± SEM; the intake was analyzed by two-way ANOVA. Different letters are significantly different: *p* < 0.05. AA: offspring born to dams fed an amino acid-based diet and weaned to an amino acid-based diet; AI: offspring born to dams fed an amino acid-based diet and weaned to an intact protein diet; IA: offspring born to dams fed an intact protein diet and weaned to an amino acid-based diet; II: offspring born to dams fed an intact protein diet and weaned to an intact protein diet; M: maternal diet; W: weaning diet; T: time; NS: not significant. AUC: area under the curve.

Birth weight was altered by maternal diet and it was higher in pups born to dams fed IPD (6.34 ± 0.08 g and 6.06 ± 0.09 g in offspring born to IPD and AAD dams, respectively) (*p* < 0.05). Body weight was also higher at weeks 13 to 18 pw in pups born to IPD dams (*p* < 0.009) (Figure 4). However, fat and lean mass were not affected by either maternal or weaning diet at week 18 pw (Figure 3b). Long-term food intake was also affected by maternal diet but not by weaning diet. It was higher in offspring born to IPD dams compared with those born to AAD dams (*p* < 0.02) (Figure 5a). Consistently, the Cal intake was affected by the maternal diet and also by the weaning diet. It was higher in offspring born to IPD dams and weaned to intact protein diet compared with those born to AAD dams and weaned to amino acid-based diet (Figure 5c). Nitrogen intake was also affected by maternal diet and also by weaning diet (Figure 5e). It was higher in offspring born to IPD dams and weaned to an intact protein diet compared with those born to AAD dams and weaned to an amino acid-based diet (Figure 5c). However, when food and Cal intake were adjusted based on BW (Figure 5b,d respectively), no effect of maternal diet was observed while they were still affected by the weaning diet. They were higher in offspring weaned to an intact protein diet compared with those weaned to an amino acid-based diet. Similarly, nitrogen intake when adjusted based on BW was not influenced by maternal diet but was higher in offspring weaned to an intact protein diet compared with those weaned to an amino acid-based diet (Figure 5f).

Short-term food intake in response to protein preloads was affected by maternal diet only in the first hour of food intake (Table 2). Food intake suppression induced by protein preloads, as demonstrated by the difference in food intake after water control and after protein preload, was stronger in pups born to IPD dams (*p* < 0.05). This effect was not influenced by either weaning diet or protein preloads. However, it was an interaction of the maternal and the weaning diet with the time. No difference in 2 and 12 h FI was observed. Fasting plasma concentrations of insulin and ghrelin were not affected by either maternal or weaning diet at birth, weaning, and at week 18 pw while plasma concentrations of PYY was influenced by maternal diet at weaning. It was higher in offspring born to IPD dams compared with those born to AAD dams (*p* < 0.05). Fasting blood glucose (FBG) was also altered by the maternal diet: it was higher in pups born to AAD dams compared with those born to IPD dams at week 18 pw (*p* < 0.05) (Table 1).

## 3. Discussion

The results of this study support the role of the characteristics of proteins fed during gestation and lactation in the regulation of food intake, intake regulatory hormones, and BW of the dams and their offspring. Moreover, these results indicate that, as we previously reported [13,14,15], the effect of proteins during development can be, at least partially, due to those individual characteristics of proteins that are structure-dependent.

The role of the chemical and physical characteristics of proteins and their effect on food intake, intake regulatory system, body weight, and body composition was studied. The effects of individual AAs, amino acid composition, and bioactive peptides encrypted in the amino acid sequences of individual proteins, plus the digestibility and digestion kinetics on the regulation of food intake and body weight, have been examined separately in previous studies.

Both the amino acid- and intact protein-based diets used in this study are standardized diets (AIN-93 G-based diets) and are nutritionally adequate and balanced. Although the difference in their amino acid composition and content is negligible, the lysine content is exceptionally higher (~20%) in AAD compared with the IPD (Table 4). The effect of lysine on food intake, food efficiency, and body weight has been studied. In one study, growing rats fed a high lysine-free transgenic rice or rice supplemented with lysine showed improved growth and increased food efficiency and lysine availability compared with those fed a wild-type rice [33]. However, the comparison was between wild-type rice which is limited in lysine as an indispensable amino acid with rice supplemented with lysine and transgenic rice enriched with lysine. In another study, a higher valine/lysine ratio maximized growth rate in sows [34]. Moreover, a lower lysine/methionine ratio (~2.8/1) in the diet supplemented with methionine during late gestation increased food intake and plasma insulin concentration in dairy cows and birth weight in their offspring [35]. These results are consistent with our observation: a lower lysine/methionine ratio in IPD (2.79/1) compared with AAD (3.58/1) resulted in higher food intake and body weight in IPD-fed dams and also higher birth weight and body weight in their offspring. However, the underlying mechanisms are still unclear. Moreover, feeding an AAD during gestation resulted in a ~4.4% reduction in offspring’s birth weight, which would be termed intrauterine growth restriction (IUGR) in humans. Therefore, although nitrogen intake was higher in IPD-fed dams compared with AAD-fed dams during gestation, these results can also be interpreted as a programming effect of IUGR rather than an effect of the nature of nitrogen intake per se.

Higher caloric intake in AAD-fed dams and their offspring was not just due to their greater amount of the diet consumed. Relatively higher calorie content of the AAD (in a 100 g diet) compared with the IPD (5.9%) can also be a contributing factor in the higher calorie intake observed in AAD-fed dams and their offspring, and consequently higher body weight. Although it did not affect body composition in offspring at week 18 pw.

Although maternal diet influenced food, calorie, and also nitrogen intake, this effect was diminished when adjusted based on BW. However, weaning diet was still influential. It may lead to this conclusion that current body weight is the predominant player determining food, Cal, and nitrogen intake. However, the fact that body weight is mainly determined by the maternal diet may illustrate the significance of the maternal diet during gestation and lactation. Moreover, the significant effect of the weaning diet on Cal and nitrogen intake (both absolute and adjusted values) and also its relatively significant effect on BW (*p* = 0.06) in the post-weaning period may indicate that the diet during early life may also play a major role in determining food intake and body weight.

Moreover, the difference in digestion kinetics between free AAs in AAD and intact casein in IPD may play a role in the outcomes of this study. Casein as the sole source of protein in IPD is classified as a slow protein that is precipitated in the stomach and gradually releases AAs and peptides to the duodenum [19]. This process provides a slow but steady flow of AAs to the blood over a period of time. In contrast, the absorption of free AAs in AAD are prominently faster. This quick absorption of AAs may alter post-absorptive metabolism of AAs [3,36,37]. In one study, rapid digestion and absorption of AAs from soy protein, as a fast protein, resulted in an increased rate of amino acid degradation that eventually caused lower protein synthesis compared with casein as a slow protein [38]. It may contribute to lower lean mass and body weight observed in dams fed AAD. However, whether the difference in digestion kinetics is a factor influencing the fetal and post-natal development by altering amino acid flow in the placenta and fetus or by altering amino acid composition and concentration of milk during lactation is still unclear and needs further study.

Short-term food intake in response to protein preloads was influenced by maternal diet but not by either weaning diet or protein preload at first hour of food intake and rats born to IPD dams have shown stronger food intake suppression in response to protein preloads compared with those born to AAD dams. However, the interaction between weaning diet and maternal diet over time may indicate that the effect of the weaning diet might be masked by the effect of the maternal diet and it may have more significant effect over a longer period of time. We previously reported that the source of protein in the maternal diet altered short-term food intake in response to protein preloads along with alteration in food intake suppression induced by cholecystokinin (CCK) and also hypothalamic gene expression of agouti-related protein (AgRP) receptors [14]. The mechanisms by which maternal diet altered food intake suppression induced by protein preloads at 1 h in this study is not clear, but peripheral and hypothalamic intake regulatory hormones are potential factors.

Although no difference in the effect of preloads on food intake suppression was observed and intact protein and amino acid mixed preloads suppressed food intake at 1, 2, and 12 h similarly, the underlying mechanisms can be different. Bioactive peptides encrypted in amino acid sequences of intact casein as well as the effect of individual AAs on appetite and food intake are attributing factors to food intake suppression induced by intact casein. Moreover, several peptides released from casein during the digestion process that express a variety of functions in the gastro-intestinal (GI) tract including release and activation of digestive enzymes, nutrient absorption process, and also post-absorptive metabolic signaling pathways [39,40,41]. Although the satiety effect of BAPs released from casein is evident, as previously mentioned, no difference in short-term food intake in response to amino acid mixed and intact protein preloads at 1 and 2 h was observed. It could be due to: (1) casein being a slow protein and releasing peptides and AAs gradually; and (2) the satiating effect of individual AAs presented in amino acid-mixed preload may contribute to short-term food intake suppression induced by amino acid-mixed preload since these AAs are readily available in the GI tract. Food intake suppression induced by leucine through serotonin has been shown [19]. Although the leucine content of both preloads is very similar, the variation in digestion kinetics of free leucine in amino acid-mixed preloads and leucine encrypted in casein molecules diverge on their effect on short-term food intake consistent with our observations in the first hour of food intake in this study. Moreover, rapid digestion and absorption of AAs from amino acid-mixed preload may result in a higher plasma concentration of AAs leading to higher amino acid residue from the protein synthesis process that may in turn act as satiety signals based on the amino-static theory suggested by Mellinkoff [18]. Therefore, the difference in digestion kinetics of intact casein and amino-acid mixed preload is crucially important in the difference in the first hour of food intake observed in this study. Moreover, maternal diet did not influence fasting plasma concentrations of intake regulatory hormones (ghrelin, PYY, and insulin) at birth, weaning, and at week 18 post-weaning with the exception of fasting PYY, which was higher in offspring born to IPD dams at weaning. However, the difference disappeared at week 18 post-weaning which is consistent with similar food intake suppression induced by amino acid-mixed and intact protein preloads at week 18 post-weaning.

Food intake in the long term was influenced by maternal diet in both mothers and offspring, and was lower in maternal AAD groups. However, the mechanism by which maternal diet influenced food intake in mothers could be entirely different from those influencing food intakes in offspring. Lower food intake in the AAD-fed dams could be due to the effect of lower lysine/methionine ratios in AAD. In offspring, while no effect of weaning diet on long-term food intake was observed, those born to dams fed AAD had lower food intake compared with those born to dams fed IPD. The difference in the effect of IPD and AAD on metabolic fate of proteins in mothers and also on the amino acid flow in the placenta and eventually the development of food intake and body weight regulatory systems in fetus can be attributed to the difference in the digestion kinetics of IPD and AAD and also the existence of bioactive peptides (BAPs) in IPD and their absence in AAD that needs more study.

Birthweight was lower in offspring born to dams fed AAD compared with IPD, while both diets were nutritionally adequate. In spite of the significance of the birthweight as a factor predicting future risk of chronic diseases, the cause is not clear at this point. Higher lysine/methionine ratio in maternal AAD compared with IPD may contribute to lower food intake and body weight in AAD-fed dams and also lower birth weight and body weight in their offspring. We believe the difference in birthweight observed in this study is crucially important for future research to examine the effect of the characteristics of the proteins and to determine the potential mechanisms by which these characteristics may influence developmental programming. We previously reported that offspring born to dams fed AAD had higher systolic blood pressure and fasting blood glucose compared with those born to dams fed IPD at weeks 16 and 18 post-weaning, respectively [19], which is consistent with previous studies demonstrating a strong correlation between lower birthweight and higher risk of glucose intolerance and hypertension [42,43]. A rapid catch-up growth observed in offspring born to dams fed AAD following lower birthweight is consistent with previous studies [44,45,46,47,48]. Moreover, in spite of lower birthweight and lower body weight in offspring born to dams fed AAD at week 18, no difference in fat/weight% was observed at week 18, which is consistent with previous studies [49]. Nonetheless, the fact that both diets are standard and are nutritionally balanced support this concept that there are parameters beyond nutritional properties of dietary proteins that are determining the phenotype of the offspring.

While the difference in characteristics of maternal intact protein- and amino acid-based diets altered the phenotype of offspring, it did not have any impact when fed as a weaning diet. In contrast, it altered food intake and body weight in dams in the post-partum period. Whether the effects of the weaning diets were concealed by the effects of the maternal diets and may show some impact over a longer period of time needs further study.

In summary, the results of this study for the first time support the notion that the chemical and physical characteristics of proteins are potential factors influencing food intake and body weight in offspring. However, this area of research is still in the primary stages and much more research is needed to reveal the underlying mechanisms by which proteins and AAs influence development during pregnancy and lactation. Altering the protein and amino acid content of maternal diets during gestation does not necessarily lead to alteration in amino acid supply to the fetus. Similarly, altering the maternal diet during lactation does not necessarily change the milk composition. Measuring umbilical blood amino acid concentration, determining amino acid composition and concentration in mothers’ milk and in offspring through neonatal gastrostomy, and also evaluating hepatic amino acid metabolism and protein synthesis can help to understand underlying mechanisms.

## 4. Materials and Methods

### 4.1. Animals and Diets

First-time pregnant Wistar rats were received at day 3 of gestation (Charles River, NC, USA) and housed individually in ventilated plastic cages with bedding at 22 °C ± 1 °C and 12-h light–dark cycle (lights off at 0900 to 2100 h). The diets and water were provided ad libitum throughout the experiment. Access to the diet was limited to 12 h dark period only during the short-term food intake measurement at week 18 post-weaning.

Diets (AIN-93G) were purchased from Dyets (Dyets Inc., Bethlehem, PA, USA). The composition (per kilogram diet) of the diets and the amino acid composition of the intact protein (casein) and the amino acid mix are illustrated in Table 3 and Table 4, respectively. The protocol was approved by the University of North Florida Institutional Animal Care and Use Committee (IACUC, protocol No: IA 13-017).

### 4.2. Experimental Design

Newly pregnant Wistar rats (*n* = 24) were received on the third day of pregnancy (the whole duration of the pregnancy of the rats was approximately 21 days) and were allocated to two groups (*n* = 12/group). Rats received either an amino acid- (AAD) or an intact protein-based diet (IPD) during pregnancy and lactation. BW and food intake (FI) of the dams were measured weekly. Body weight of the pups was measured at birth (day 1, after litters were culled to 10 pups/dam) and on days 7, 14, and 21. At weaning (day 21 of age), one male offspring from each dam on each maternal diet group was assigned to either the AAD or IPD (*n* = 12/group). Body weight and FI were measured weekly for 18 weeks after weaning. Fasting plasma concentration of ghrelin, Polypeptide Y (PYY), insulin, and glucose (FBG) were measured at week 6 post-weaning (pw) for the dams and at birth, weaning and at week 18 pw for the offspring. Fat pad mass was measured at killing at week 6 pw for the dams and at week 18 pw for offspring. Short-term food intake was measured at week 18 post-weaning.

### 4.3. Long- and Short-Term Food Intake

Long-term food intake was measured on a weekly basis by weighing the food containers at the beginning and at the end of each week. Spillage was collected, measured, and subtracted to obtain the actual intake. Calorie intake was calculated based on the calorie content of the diets and the amounts consumed. Nitrogen intake, as an indicator of protein intake, was also calculated based on the protein content of the diets and the amounts of protein consumed.

Short-term food intake was also measured in pups at week 18 pw. After an overnight fast, food intake at 1, 2, and 12 h was measured following protein preloads. During an adaptation process (7 days), rats were gavaged with water (4 days) and their food was removed for 12 h. On day 5, half of the rats received the gavage while the rest were untreated. On day 6 of adaptation, the testing order was reversed. Only when it was determined that there was no effect of gavage on actual FI, the experiment started.

### 4.4. Protein Preloads 

Intact casein and amino acid mix were obtained from Dyets (Dyets Inc., Bethlehem) (Table 4). Each rat received 3 g/kg BW of either intact casein or amino acid mix per 6 mL distilled water by gavage 30 min prior to introducing the food and after 12 h fasting. Administration of protein preloads can help to understand how proteins may influence food intake in short-term differently and in a time-dependent manner.

### 4.5. Blood Glucose

Tail vein glucose concentration was assayed using a handheld commercial glucometer (Contour^®^ Next Blood Glucose Meter, Bayer Healthcare LLC, Mishawaka, IN, USA) using test strips. The accuracy and variance of the glucometer and test strips were examined by applying control solutions (levels 1 and 2) provided by the manufacturer (Bayer, Bayer Healthcare LLC).

### 4.6. Blood Collection

As previously described [13], trunk blood was collected in chilled vacutainer tubes (BD Diagnostic, Franklin Lakes, NJ, USA) containing EDTA + Trasylol® (Bayer AG, Leverkusen, Germany) solution (10% blood volume, 5 × 10^8^ Iu/L). Blood samples were centrifuged at 3000 g at 4 °C for 10 min afterward. Plasma was separated and immediately stored at −70 °C.

### 4.7. Hormone Assays

Enzyme-linked immunosorbent assays (ELISAs) were used to measure plasma concentrations of PYY (catalog no. 48-PYYRT-E01.1, Alpco Diagnostics, Salem, NH, USA), ghrelin (catalog no. 32-5118, Alpco Diagnostics), and insulin (catalog no. 80-INSRT-E01, Alpco Diagnostics). Assay sensitivity for PYY, ghrelin, and insulin was 0.31 ng/mL, 0.8 pg/mL, and 0.124 ng/mL, respectively.

### 4.8. Body Composition

Fat mass (FM) and lean mass were measured right after killing at weeks 6 and 18 pw for dams and pups, respectively. Fat mass was measured by dissection of extracted abdominal, epididymal, and perirenal fat [13]. Therefore, lean mass described here also includes fat in other areas of the body particularly subcutaneous fat.

### 4.9. Statistical Analyses

The effect of the maternal and weaning diets and their interactions on BW and short- and long-term FI was analyzed by two-way ANOVA. When repeated measures were made over time on BW and FI, the Mixed procedure was used with maternal diets, weaning diets, and time as the main factors. When interactions were statistically significant, a one-way ANOVA followed by post-hoc Tukey’s test was conducted to evaluate treatment effects. The effects of the maternal diets on plasma measures including FBG, insulin, ghrelin, and PYY were compared using a Student’s unpaired *t*-test. Data are expressed as means with standard errors. Statistical significance was defined at *p* < 0.05. All analyses were conducted using SAS (version 9.4; SAS Institute, Cary, NC, USA).

## 5. Conclusions

The result of this study supports the role of the characteristics of dietary proteins and their effect on body weight and development of a food intake regulatory system. While maternal diet influenced food, calorie, and nitrogen intake and body weight in both dams and offspring, weaning diet showed a minimal effect. However, the effect of the weaning diet on food, calorie, and nitrogen intake was significant when they were adjusted based on body weight. Moreover, the fact that both the AAD and IPD diets are nutritionally balanced and complete diets may raise the notion that the metabolic and physiologic responses to proteins depends on factors that have not been esteemed in the current nutrition context. Therefore, further investigation of parameters determining metabolic and physiologic responses to dietary proteins particularly in developmental programming and incorporating this knowledge in the current nutrition context is crucial and needs an abrupt attention.

## Figures and Tables

**Table 1:** Effect of maternal diet on fasting plasma ghrelin, PYY, glucose, and insulin in dams at week 9 post-partum and in pups at birth, weaning, and at week 18 post-weaning. (*n* = 5–6/group). Data are means ± SEM; AAD: amino acid-based diet; IPD: intact protein diet; PYY: Peptide YY.

Maternal Diet		IPD		AAD		*p*
***Dams: At wk 9 postpartum***						
Ghrelin, pg/mL		3.73 ± 0.07		3.31 ± 0.11		NS
PYY, ng/mL		0.87 ± 0.07		0.95 ± 0.08		NS
Glucose, mM		5.31 ± 0.28		4.90 ± 0.28		NS
Insulin, ng/mL		2.28 ± 0.43		2.35 ± 0.65		NS
***Pups: At Birth***						
Ghrelin, pg/mL		2.00 ± 0.73		2.61 ± 0.40		NS
PYY, ng/mL		1.02 ± 0.05		1.17 ± 0.15		NS
Glucose, mM		5.25 ± 0.78		5.36 ± 0.38		NS
Insulin, ng/mL		1.13 ± 0.30		1.18 ± 0.14		NS
***Weaning***						
Ghrelin, pg/mL		3.62 ± 0.10		3.78 ± 0.06		NS
PYY, ng/mL		1.08 ± 0.07		0.79 ± 0.05		*p *< 0.05
Glucose, mM		5.41 ± 0.50		4.88 ± 0.56		NS
Insulin, ng/mL		1.66 ± 0.06		1.91 ± 0.24		NS
***At wk 18 PW***						
Ghrelin, pg/mL		9.55 ± 0.97		7.75 ± 1.13		NS
PYY, ng/mL		1.25 ± 0.35		1.17 ± 0.03		NS
Glucose, mM		4.64 ± 0.15		5.52 ± 0.20		*p *< 0.05
Insulin, ng/mL		2.17 ± 0.42		3.32 ± 0.10		NS

**Table 2:** Effect of maternal diet on food intake in response to water and protein preloads at 1, 2, and 12 h in male offspring at week 18 pw.

Maternal Diet			IPD					AAD			
Weaning Diet	IPD			AAD		IPD			AAD		
	Intact	AA-based		Intact	AA-based	Intact	AA-based		Intact	AA-based	
	Casein	Casein		Casein	Casein	Casein	Casein		Casein	Casein	*p*
0–1 h											
Control (water)	7.18 ± 0.62			6.23 ± 0.87		5.70 ± 0.47			6.77 ± 0.88		M: *p* = 0.05
Protein	4.47 ± 0.67	3.68 ± 0.61		6.48 ± 0.85	5.17 ± 0.43	3.30 ± 0.81	3.80 ± 0.60		4.15 ± 0.30	5.30 ± 0.33	W: NS
Water-Protein	2.77 ± 0.42 *	3.50 ± 0.44 *		0.25 ± 1.63	0.80 ± 0.21	2.80 ± 1.06 *	1.90 ± 0.50 *		2.7 ± 1.21 *	1.40 ± 1.34 *	P: NS
											T: *p* < 0.0001
0–2 h											M × T: *p* < 0.05
Control (water)	5.20 ± 1.70 ^b^			10.68 ± 2.62 ^a^		7.53 ± 0.49 ^b^			6.90 ± 0.68 ^b^		M × W × T: *p* < 0.05
Protein	7.83 ± 1.97	7.08 ± 2.45		11.08 ± 1.19	9.90 ± 2.50	7.17 ± 1.96	5.13 ± 0.63		7.55 ± 1.68	6.40 ± 1.03	W × T: *p* < 0.05
Water-Protein	2.93 ± 4.14	1.88 ± 3.70		0.40 ± 1.71	1.17 ± 0.55	0.70 ± 2.31	2.40 ± 0.58		1.03 ± 2.98	1.13 ± 0.94	P × T: *p* < 0.05
0–12 h											
Control (water)	16.58 ± 0.82			20.95 ± 2.69		19.05 ± 2.35			18.83 ± 0.97		
Protein	19.30 ± 3.15	15.25 ± 2.04		22.10 ± 0.95	21.53 ± 1.92	20.10 ± 1.96	17.03 ± 1.36		20.03 ± 2.26	17.93 ± 1.42	
Water-Protein	2.07 ± 2.28	0.68 ± 2.88		1.15 ± 2.03	1.70 ± 0.85	1.57 ± 0.77	2.03 ± 1.08		1.90 ± 4.00	1.1 ± 2.06	

Values (means ± SE) are expressed in g; *n* = 11–12 per group. Protein preloads (3 g/kg body wt) were given by gavage 30 min before food was introduced. Distilled water (6 mL) was used for water (control). Water-protein was calculated as food intake after water preload-food intake after protein preload. Food intake was analyzed by MIXED Model followed by Tukey’s post hoc test, with maternal diet, weaning diet, preload and time as main factors. M: Maternal diet; W: weaning diet; P: Preload; T: Time; Values in a row with different superscript letters (a,b) are significantly different, *p* < 0.05. * Significant food intake suppression: *p* < 0.05.

**Table 3:** Amino acid composition of the intact protein- and amino acid-based AIN-93 G Diets.

	Intact Protein		Amino Acid-Mixed
	Diet		Diet
		g/kg	
Alanine	4.7		4.5
Arginine	6.5		6.3
Aspartic acid	11.7		11.3
Cystine	3.7 *		3.7
Glutamic acid	37.5		36.2
Glycine	3.2		3.1
Histidine	4.7		4.5
Isoleucine	8.6		8.4
Leucine	15.9		15.3
Lysine	13.3		16.1
Methionine	4.7		4.5
Phenylalanine	9.0		8.7
Proline	21.1		20.4
Serine	9.7		9.4
Threonine	6.8		6.6
Tryptophan	2.2		2.1
Tyrosine	9.6		9.2
Valine	10.3		9.9

**Table 4:** Composition of the intact protein- and amino acid-based AIN-93 G Diets.

	Intact Protein		Amino Acid-Mixed
	Diet		Diet
Cal	3597		3811
		g/kg	
Casein	200		0
Amino acid Mix	0		180
Sucrose	100		100
Soybean oil	70		70
t-Butyhydroquinone	0		0.014
Cornstarch	397.5		399.9
Dyetrose	132		145
Cellulose	50		50
Mineral Mix	35		35
Sodium Bicorbonate	0		7.4
Vitamin Mix	10		10
Choline Bitartrate	2.5		2.5
L-Cystine	3		0

## Abbreviations

AA	amino acid
AAD	amino acid-based AIN-93 G diet
BAPs	bioactive peptides
BG	blood glucose
BW	body weight
ELISA	enzyme-linked immunosorbent assay
FI	food intake
FM	fat mass
IPD	intact protein-based AIN-93 G diet
PW	post-weaning
PYY	peptide YY
